# Genetic and Biochemical Analysis of the *Azotobacter vinelandii* Molybdenum Storage Protein

**DOI:** 10.3389/fmicb.2019.00579

**Published:** 2019-03-21

**Authors:** Mónica Navarro-Rodríguez, José María Buesa, Luis M. Rubio

**Affiliations:** Centro de Biotecnología y Genómica de Plantas (CBGP), Universidad Politécnica de Madrid (UPM), Instituto Nacional de Investigación y Tecnología Agraria y Alimentaria (INIA), Madrid, Spain

**Keywords:** nitrogenase, iron–molybdenum cofactor, nitrogen fixation, metal homeostasis, MoSto

## Abstract

The N_2_ fixing bacterium *Azotobacter vinelandii* carries a molybdenum storage protein, referred to as MoSto, able to bind 25-fold more Mo than needed for maximum activity of its Mo nitrogenase. Here we have investigated a plausible role of MoSto as obligate intermediate in the pathway that provides Mo for the biosynthesis of nitrogenase iron–molybdenum cofactor (FeMo-co). The *in vitro* FeMo-co synthesis and insertion assay demonstrated that purified MoSto functions as Mo donor and that direct interaction with FeMo-co biosynthetic proteins stimulated Mo donation. The phenotype of an *A. vinelandii* strain lacking the MoSto subunit genes (Δ*mosAB*) was analyzed. Consistent with its role as storage protein, the Δ*mosAB* strain showed severe impairment to accumulate intracellular Mo and lower resilience than wild type to Mo starvation as demonstrated by decreased *in vivo* nitrogenase activity and competitive growth index. In addition, it was more sensitive than the wild type to diazotrophic growth inhibition by W. The Δ*mosAB* strain was found to readily derepress *vnfDGK* upon Mo step down, in contrast to the wild type that derepressed Vnf proteins only after prolonged Mo starvation. The Δ*mosAB* mutation was then introduced in a strain lacking V and Fe-only nitrogenase structural genes (Δ*vnf* Δ*anf*) to investigate possible compensations from these alternative systems. When grown in Mo-depleted medium, the Δ*mosAB* and *mosAB*^+^ strains showed low but similar nitrogenase activities regardless of the presence of Vnf proteins. This study highlights the selective advantage that MoSto confers to *A. vinelandii* in situations of metal limitation as those found in many soil ecosystems. Such a favorable trait should be included in the gene complement of future nitrogen fixing plants.

## Introduction

Nitrogenase, the enzyme complex that catalyzes the fixation of N_2_ into NH_3_, is one of the most relevant enzymes in the nitrogen cycle since it converts inert N into a biologically usable form. In its most prevalent type nitrogenase is an iron–sulfur molybdoenzyme (Boyd and Peters, 2013), although other phylogenetically related nitrogenases exist that carry iron-sulfur-vanadium or iron–sulfur only cofactors (Bishop and Joerger, 1990; Eady, 1996). The Mo-nitrogenase consists of a dinitrogenase component, a NifDK heterotetramer containing two pairs of metalloclusters named P-cluster (8Fe-7S) and FeMo-co (7Fe-9S-C-Mo-*R*-homocitrate) (Kim and Rees, 1992; Einsle et al., 2002; Rubio and Ludden, 2008; Spatzal et al., 2011), and a dinitrogenase reductase component formed by two NifH homodimers each one carrying a [4Fe-4S] cluster (Georgiadis et al., 1992). *Azotobacter vinelandii* has the peculiarity of having genes to encode the Mo-nitrogenase (*nif*) and the alternative V (*vnf*) and Fe-only (*anf*) nitrogenases (Bishop and Joerger, 1990). The dinitrogenase components of the alternative nitrogenases contain additional subunits (VnfG or AnfG) essential for N_2_ reduction (Chatterjee et al., 1997; Krahn et al., 2002) and present subtle differences in cofactor structure (Sippel and Einsle, 2017). However, amino acid sequence comparisons of NifD/VnfD/AnfD and NifK/VnfK/AnfK indicate that residues that serve as ligands to the metal cofactors are conserved in all three nitrogenases (Joerger et al., 1990).

*Azotobacter vinelandii nif*, *vnf*, and *anf* genes are differentially expressed depending on the metal availability in the environment following a hierarchical *nif* > *vnf* > *anf* sequence. The presence of as low as 50 nM molybdate in the medium represses *vnf* and *anf* genes while vanadium represses the *anf* but not the *nif* genes (Jacobson et al., 1986; Luque and Pau, 1991; Jacobitz and Bishop, 1992). As consequence V-nitrogenase is active in the absence of Mo when V is available and Fe-only nitrogenase is active when neither Mo nor V is available in the medium.

Molybdenum is an essential transition metal for most organisms, which typically carry a number of enzymes and proteins involved in its uptake, storage, homeostasis, regulation, and Mo cofactor biosynthesis (Hernandez et al., 2009; Hille et al., 2014). This metal, and its biological antagonist tungsten (W), can exist in several oxidation states ranging from -II to +VI, with MoO_4_^2-^ being the main source of Mo at neutral and basic pH. However, Mo availability in soil ecosystems depends on pH, reactive oxides and water drainage and it is often a limiting factor for nitrogen fixation (Reddy et al., 1997).

Bacteria have developed high-affinity chelation and uptake mechanisms to scavenge molybdate from the environment, including metallophores, ABC-type transporters, and Mo storage proteins (Mouncey et al., 1995; Kraepiel et al., 2009). *A. vinelandii* carries an unusual Mo-binding protein called molybdenum-storage protein (MoSto) (Pienkos and Brill, 1981; Fenske et al., 2005), a (αβ)_3_ hexameric complex encoded by the homologous *mosA* and *mosB* genes that is capable of binding more than 100 Mo atoms in the form of polyoxomolybdate clusters (Schemberg et al., 2007; Kowalewski et al., 2012; Poppe et al., 2014). MoSto can also accumulate W (Schemberg et al., 2007). Metal binding to MoSto requires ATP hydrolysis while metal release is ATP-independent but pH-dependent occurring stepwise above pH 6.8 (Schemberg et al., 2008). No amino acid sequence to other Mo-containing enzymes has yet been described. MoSto structure has been related to nucleoside monophosphate kinases, particularly with the UMP kinase from bacteria and archaea, which uses ATP to phosphorylate UMP (Ramon-Maiques et al., 2002; Poppe et al., 2014).

*Azotobacter vinelandii* is known to accumulate 25-fold more Mo than required for maximum nitrogenase activity (Shah et al., 1984) probably due to the presence of MoSto. However, the effect on nitrogenase of eliminating MoSto has never been determined. The aims of this study are the phenotypical characterization of a MoSto deficient strain and the elucidation of MoSto involvement in FeMo-co biosynthesis.

## Results

### MoSto Serves as Mo Donor for *in vitro* FeMo-co Biosynthesis

**FIGURE 1:**
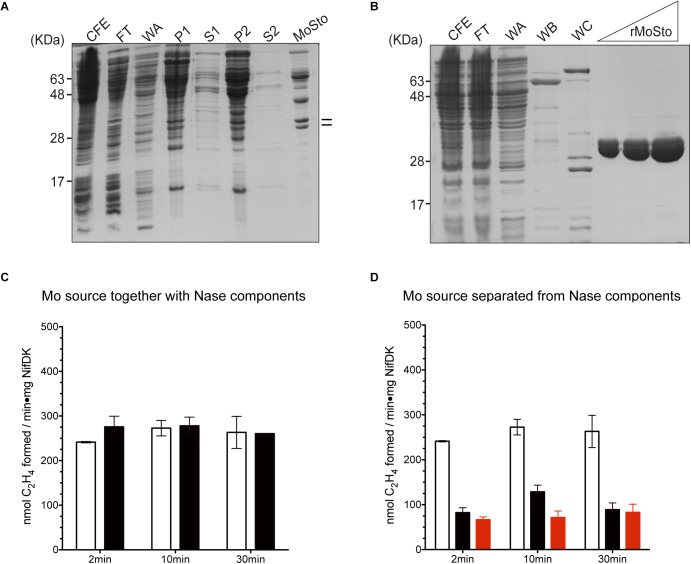
*In vitro* FeMo-co synthesis and nitrogenase reconstitution time-course assays using MoSto as source of Mo. **(A)** Partial purification of MoSto from *Azotobacter vinelandii* cells. Lines indicate migration of MoSto subunits. **(B)** Purification of rMoSto from *Escherichia coli* cells. CFE, cell-free extract; FT, first column flow through; WA, WB, and WC column washes with increasing imidazole; P and S, pellet and supernatant of ammonium sulfate fractionations. **(C)** Reactions in which the molybdenum source (molybdate or MoSto) is mixed with the FeMo-co biosynthetic proteins. Molybdenum sources used were: 7.5 μM molybdate (white bars) or MoSto protein equivalent to 4.5 μM Mo (black bars). **(D)** Reaction mixtures in which the molybdenum source: molybdate (white bars), MoSto (black bars), or rMoSto (red bars) is separated from FeMo-co biosynthetic proteins by a dialysis membrane. Molybdenum sources used were: 7.5 μM molybdate, 22.8 μg MoSto (4.5 μM Mo), or 229 μg rMoSto (11 μM Mo). Data represent mean ± standard deviation of three independent experiments.

Two versions of MoSto were purified to test their capacity to serve as Mo donor in the *in vitro* FeMo-co synthesis assay. A non-tagged version was partially purified from cells of *A. vinelandii* DJ (Figure 1A) whereas a histidine-tagged version (hereinafter named rMoSto) was cloned, overexpressed, and purified from recombinant *Escherichia coli* cells (Figure 1B). As purified from *A. vinelandii*, MoSto carried 25-fold more Mo than rMoSto purified from *E. coli* cells grown in medium supplemented with 1 mM molybdate (Table 1). When purified from cells grown in not supplemented medium, rMoSto contained very little Mo, consistent with BL21 deficiency in high-affinity molybdate transport (Pinske et al., 2011). Both MoSto and rMoSto functioned as sole Mo source for *in vitro* FeMo-co synthesis, with both versions being equally efficient when normalized by Mo content (Table 1). Nitrogenase reconstitution levels correlated with Mo contents in all MoSto preparations. Direct interaction with FeMo-co biosynthetic proteins stimulated Mo donation by MoSto (Figure 1C). When MoSto (or rMoSto) was separated from the Nif proteins by a dialysis membrane threefold lower nitrogenase reconstitution levels were obtained (Figure 1D). These basal levels of reconstitution were probably due to Mo release from MoSto at pH 7.5 of the reaction mixture (Schemberg et al., 2008). Interestingly, reconstitution due to unspecific Mo release did not increase over time. These biochemical assays established a role for MoSto as direct Mo donor for FeMo-co synthesis.

**Table 1:** Activity of nitrogenase reconstituted with MoSto as Mo donor in the *in vitro* FeMo-co synthesis assay.

MoSto type	Mo in growth medium (mM)^a^	Mo atoms in MoSto^b^	μg MoSto used (μM Mo in reaction)	% Activity^c^
rMoSto	–	0.05 ± 0.00	10 (<0.01)	2.3 ± 0.8
			40 (0.02)	4.5 ± 0.3
rMoSto	1	4.12 ± 1.18	10 (0.48)	34.3 ± 3.2
			40 (1.94)	78.7 ± 4.6
MoSto	0.01	104.14 ± 19.8	0.5 (0.99)	55.3 ± 4.9
			1.0 (1.98)	63.1 ± 6.3


*^a^Cells used to purify MoSto were grown in media containing the indicated amount of molybdate. ^*b*^Per purified MoSto hexamer. ^*c*^Percentage of activity compared to control reactions containing 7.5 μM molybdate as Mo source. Molybdate containing reactions formed 475 nmol of C_*2*_H_*4*_ per minute per mg of MoFe protein. Data represent mean and standard deviations of three independent experiments.*

### The Absence of MoSto Impairs Molybdenum Accumulation and *in vivo* Nitrogenase Activity

**FIGURE 2:**
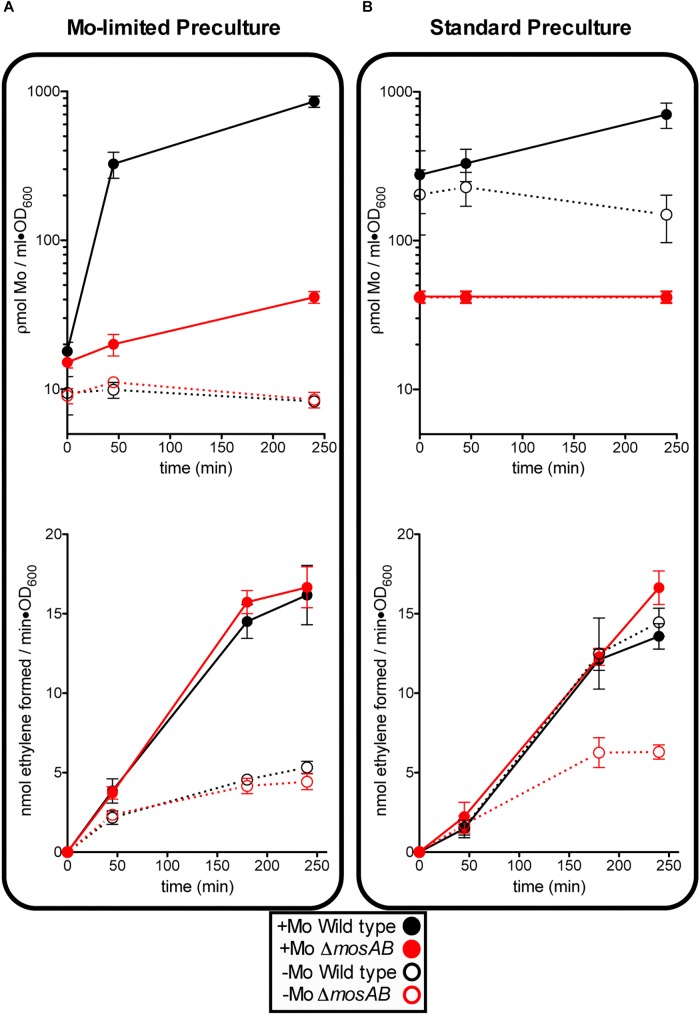
Effect of MoSto on molybdenum accumulation and *in vivo* nitrogenase activity in *A. vinelandii*. Mo-Starved **(A)** or Standard grown **(B)** cells of *A. vinelandii* DJ (wild type; black symbols) or UW394 (Δ*mosAB*, red symbols) were inoculated into Burk’s modified N-limited medium containing (close symbols) or lacking (open symbols) 1 μM molybdate. All media contained 1 μM vanadate. Data represent mean ± standard deviation of three independent experiments.

An *A. vinelandii* strain with an in-frame deletion of MoSto-encoding genes (*ΔmosAB)* was generated to investigate cellular molybdenum levels and nitrogenase activity dependence on MoSto. Four culture conditions reflecting different levels and lengths of Mo starvation were tested. On one hand, precultures obtained by subculturing at least three times in Mo-limited Burk’s modified medium (hereinafter called Mo Starved) were inoculated into N-free Mo-limited medium (-Mo) or into N-free Mo-standard medium (+Mo) (Figure 2A). On the other hand, precultures grown in Burk’s modified medium (hereinafter called Mo Standard) were inoculated into N-free Mo-limited medium (-Mo) or into N-free Mo-standard medium (+Mo) (Figure 2B). To minimize nitrogen stress all testing growth media contained 1 μM vanadate to allow V-nitrogenase synthesis at low Mo concentrations.

The following observations could be made: (*i*) In the presence of MoSto, Mo uptake by Mo-starved cells occurred much faster and maximum Mo accumulation was 100-fold higher; (*ii*) Mo content in cells lacking MoSto did not always correlate with nitrogenase levels (Figure 2A,B) suggesting the presence of alternative Mo sinks or reservoirs; (*iii*) MoSto was not required for maximum nitrogenase activity as long as Mo was provided in the growth medium indicating that its role in Mo-nitrogenase is not essential; (*iv*) However, under transient Mo starvation, tested by derepressing standard precultures in Mo-limited medium, the MoSto mutant was impaired in nitrogenase activity while the wild type was not (compare activity open circles of Figure 2A,B). This indicates that metal accumulated at MoSto is readily accessible for FeMo-co synthesis allowing maximum nitrogenase levels under transient Mo-limiting conditions. (*v*) Prolonged Mo starvation equally affected Mo content and nitrogenase activity in wild type and MoSto mutant; (*vi*) 30% nitrogenase activity remained in both strains after prolonged Mo starvation (Figure 2A) suggesting V-nitrogenase was being expressed.

**FIGURE 3:**
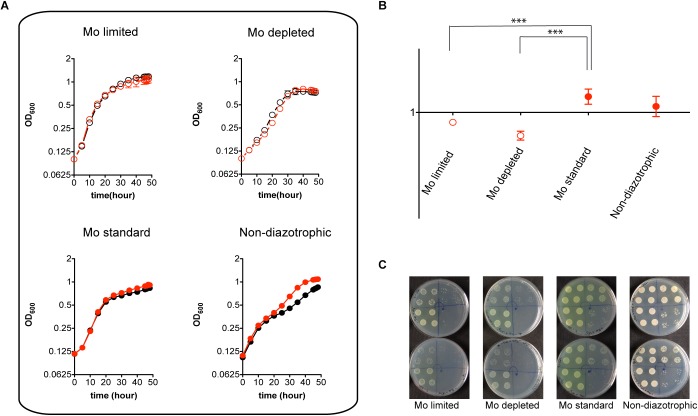
Diazotrophic growth and CI analysis of the MoSto mutant. **(A)** *A. vinelandii* DJ (wild type; black symbols) and UW394 (Δ*mosAB*, red symbols) were cultured in Burk’s modified N-free medium supplemented (close symbols) or not supplemented (open symbols) with 1 μM molybdate. Mo-limited (4 nM), Mo-depleted and Mo standard (1 μM) conditions were tested. All N-free media contained 1 μM vanadate. Growth in Burk’s modified medium containing ammonium acetate (non-diazotrophic) was carried out as control. **(B)** CI values were obtained from mixed cultures (1:1 inoculum ratio) of DJ and UW394 strains. Data represent the mean and standard deviation of three or four independent experiments. ^∗∗∗^ Indicates unpaired *t*-test *P*-value < 0.001. **(C)** Plates showing growth on solid media of mixed cultures that were pre-grown in liquid medium for 22 h. Four 20-μl drops with different dilutions (10^3^, 10^4^, 10^5^, and 10^6^) of the mixed culture were plated on each quarter of the plate. Arrows show the direction of the dilutions. Upper plates contain media lacking spectinomycin (to allow DJ and UW394 growth) whereas lower plates contain media supplemented with spectinomycin (to only allow UW394 growth).

Despite its pronounced impairment in Mo accumulation and nitrogenase activity, the MoSto mutant was only mildly affected in diazotrophic growth in Mo-limited conditions. This is consistent with the Mo requirements for maximum nitrogenase activity being much lower than the capacity of *A. vinelandii* to scavenge and accumulate Mo (Shah et al., 1984). In contrast, the MoSto mutant showed slightly better growth than wild type in the presence of 1 μM molybdate both under diazotrophic and non-diazotrophic conditions (Figure 3A). Competitive index (CI) assays were carried out to analyze the Δ*mosAB* phenotype in situations of competition for limiting Mo that are of environmental importance. In these assays growth interference takes place between the wild type and mutant strains. A CI > 1 indicates that the mutant is more competitive than wild type whereas a CI < 1 indicates the mutant is less competitive. Figure 3B,C shows that the MoSto mutant is less competitive than wild type in diazotrophic growth under Mo starvation. CI was lowest under severe Mo starvation imposed by continuous growing in Mo-depleted medium consistent with a MoSto role as Mo reservoir. Interestingly the MoSto mutant was more competitive than wild type in the presence of 1 μM molybdate in the medium. Because MoSto is present at similar levels in both conditions (see below) this result suggests that the energy burden of loading Mo into MoSto is not negligible.

### MoSto Protects *A. vinelandii* Nitrogenase From W Toxicity

**FIGURE 4:**
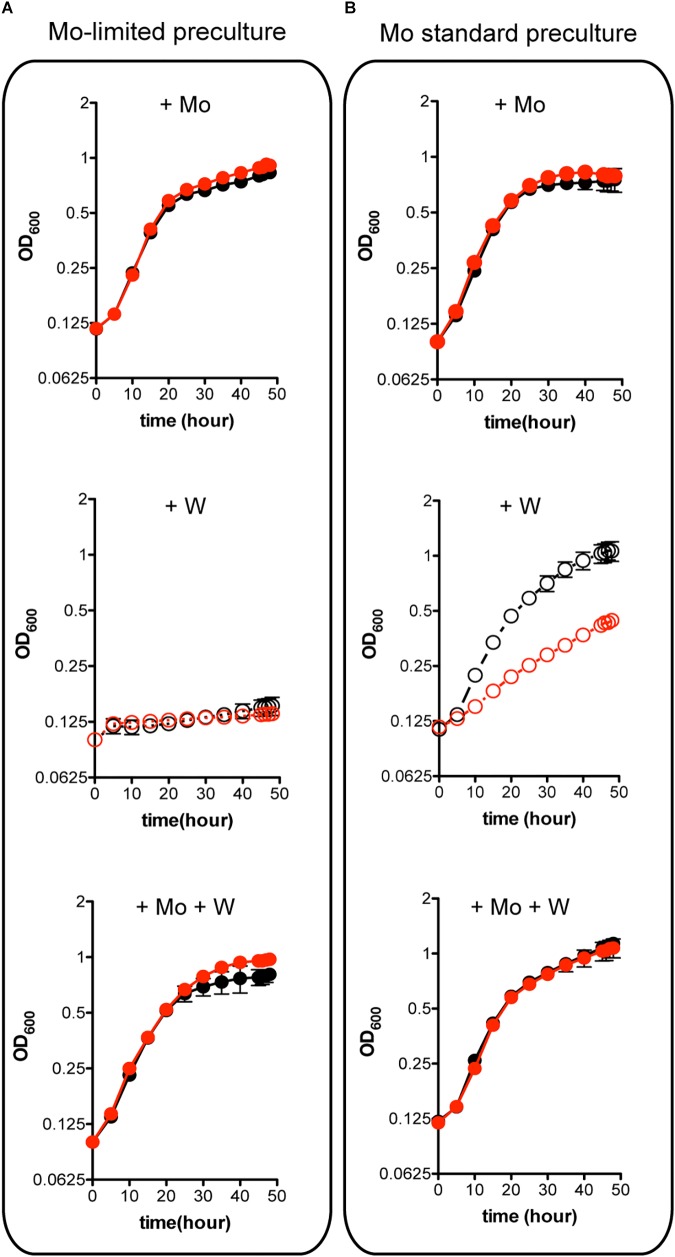
Effect of W on the diazotrophic growth of an *A. vinelandii* *ΔmosAB* strain. Mo-starved **(A)** or Mo standard **(B)** precultures of *A. vinelandii* DJ (wild type; black symbols) or UW394 (Δ*mosAB*, red symbols) were inoculated into Burk’s modified N-free medium containing 1 μM molybdate (close symbols) or 4 nM molybdate (open symbols). One μM tungstate was included when indicated and all media contained 1 μM vanadate. Data represent mean ± standard deviation of three independent experiments.

W is a well-known competitive inhibitor of Mo functions having negative effect on *A. vinelandii* growth (Keeler and Varner, 1957; Shah et al., 1984). Importantly, binding of W to MoSto has been shown to occur *in vivo* (Pienkos and Brill, 1981) and *in vitro* (Schemberg et al., 2007). The possible role of MoSto in protection against W toxicity was investigated by comparing Δ*mosAB* diazotrophic growth to that of the wild type at environmentally relevant metal concentrations (Figure 4). Mo starved precultures of both wild type and MoSto mutant were highly sensitive to inhibition by 1 μM W (a W/Mo ratio of 250) (Figure 4A) an amount that could exceed the W trapping capacity of MoSto. In contrast, when cells grown in Mo standard conditions were transferred to Mo-limited conditions in the presence of W the wild type strain grew normally while the MoSto mutant was clearly inhibited (Figure 4B). The differential behavior of wild type and the Δ*mosAB* mutant under transient Mo starving conditions can be rationalized considering MoSto as reservoir that continuously provides Mo for FeMo-co synthesis thus avoiding W toxicity. In all cases, co-presence of 1 μM molybdate in the medium protected from 1 μM tungstate inhibition whereas 1 μM vanadate did not.

### Deletion of *mosAB* Genes Affects V-Nitrogenase Accumulation Under Mo Deficiency

**FIGURE 5:**
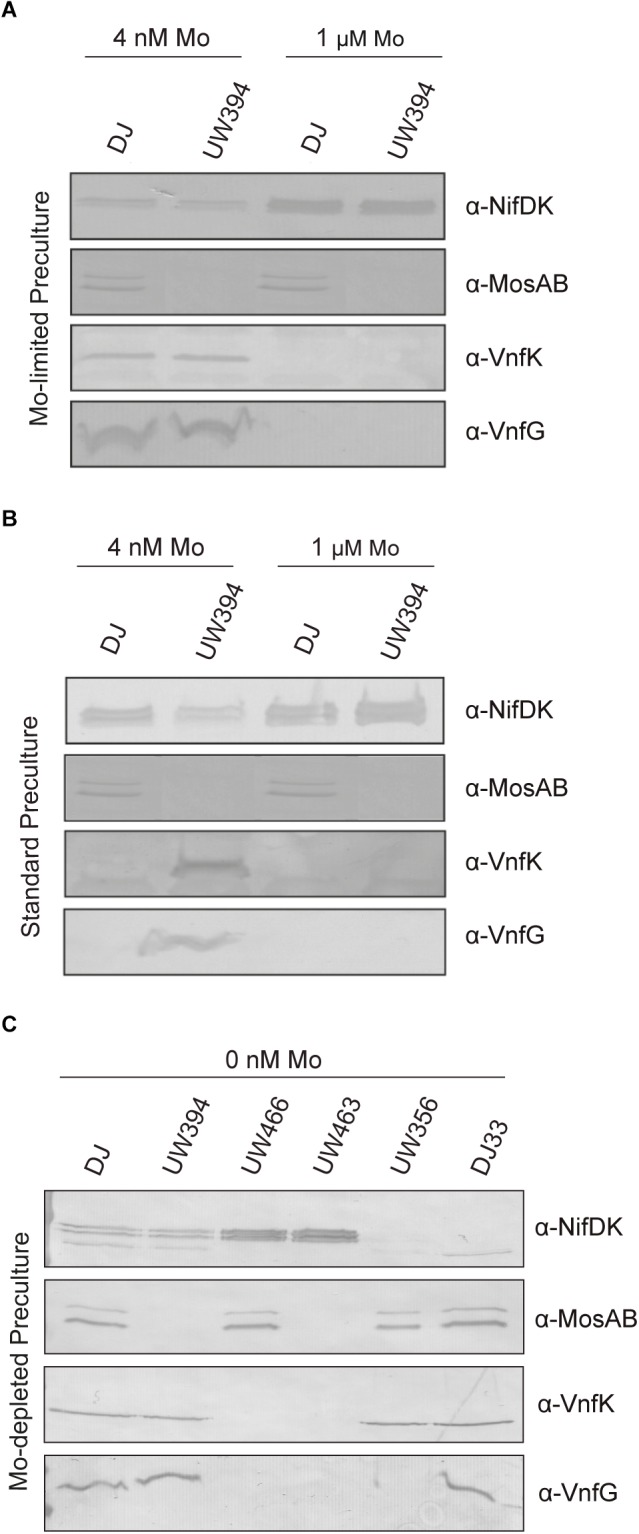
Detection of Mo-nitrogenase and V-nitrogenase structural polypeptides in *A. vinelandii*. Mo-starved **(A,C)** or Mo standard **(B)** precultures of *A. vinelandii* DJ (wild type), UW394 (Δ*mosAB*), UW466 (Δ*vnf* Δ*anf*), UW463 (Δ*vnf* Δ*anf* Δ*mosAB*), UW356 (Δ*nifA*), or DJ33 (Δ*nifDK*) were inoculated into Burk’s modified N-free medium containing either 0, 4 nM or 1 μM molybdate to allow nitrogenase derepression. NifDK, VnfK, VnfG, and MosAB polypeptides accumulated in the cells were detected by Western blot analysis against specific antibodies.

Mo tightly represses expression of V-nitrogenase (Bishop and Joerger, 1990). However, V-nitrogenase and Mo-nitrogenase transcripts coexist when molybdate levels in the medium are in the range of 10–50 nM (Jacobson et al., 1986). Because the presence or absence of MoSto largely determines intracellular Mo concentration, the effect of Δ*mosAB* mutation on the accumulation of Mo-nitrogenase and V-nitrogenase structural components in relation to changes in Mo availability was investigated. On one hand, Mo starved cells were transferred to the same Mo-limiting conditions (4 nM Mo) or to medium with standard molybdate (1 μM Mo). Standard medium allowed maximum NifDK accumulation and repressed VnfDGK synthesis, as expected (Figure 5A). Under Mo-limiting conditions, Nif polypeptides were present at much lower levels and coexisted with Vnf polypeptides, both in the wild type and in the Δ*mosAB* strain. On the other hand, transferring Mo sufficient cells to Mo-limited medium (Mo step down) readily derepressed *vnfDGK* expression in the Δ*mosAB* strain but not in the wild type, which in contrast accumulated more NifDK than the mutant (Figure 5B). Importantly, MoSto was accumulated to similar levels under all Mo concentrations tested in this study, in agreement with previous observations (Pienkos and Brill, 1981; Fenske et al., 2005). These results indicates that the buffering effect that MoSto has in Mo homeostasis (Figure 2B) softens the regulatory response to transient Mo limitation repressing early *vnf* expression and maintaining higher Nif-dependent nitrogenase activity.

### The *mosAB* Deletion Has No Effect on the Activity of Alternative Nitrogenases

The Δ*mosAB* mutation was also introduced into a Δ*vnfDGK* Δ*anfDGK* strain, lacking the V and Fe-only nitrogenase structural components, to investigate a possible role in these alternative systems. Cells cultured in Mo-depleted medium were derepressed for nitrogenase in either Mo-depleted or Mo-standard media supplemented with 1 μM vanadate. *In vivo* nitrogenase activities were estimated by measuring acetylene reduction into ethylene and ethane (Table 2), an exclusive property of alternative nitrogenases (Dilworth et al., 1987) while the presence of Mo- or V-nitrogenase structural components under Mo-depleted conditions was determined by immunoblot analysis (Figure 5C). Importantly, all Mo-depleted strains contained less than 0.7 pmol Mo per ml of culture at OD_600_ of 1 at 8 h of derepression.

**Table 2:** Ratio of ethane to ethylene production in MoSto deficient strains derepressed in media with different metal availability.

Strain^a^	Metal	C_2_H_4_^b^	C_2_H_6_/C_2_H_4_ (%)^c^
DJ	V Mo, V	1.58 ± 0.07 13.65 ± 0.55	0.63 0
UW394	V Mo, V	1.57 ± 0.01 15.24 ± 1.04	0.71 0
UW466	V Mo, V	1.83 ± 0.28 13.94 ± 1.03	0.65 0
UW463	V Mo, V	1.92 ± 0.18 14.95 ± 1.04	0.60 0
UW356	V Mo, V	0.47 ± 0.01 0	1.00 0
DJ33	V Mo, V	1.64 ± 0.59 0	1.22 0


*^a^Precultures grown in *NH*_*4*_^+^-containing Mo-depleted medium were transferred to N-free medium supplemented with 1 μM of the indicated metal. ^*b*^Activities are in nmol ethylene per min per ml at an OD_*600*_ of 1. Data represent mean and standard deviations of three independent experiments. ^*c*^C_*2*_H_*6*_ production shown as % of C_*2*_H_*4*_.*

Strains with intact Mo-nitrogenase structural genes produced maximum levels of ethylene and repressed alternative nitrogenase structural proteins in media containing 1 μM Mo and 1 μM V, as expected. Under Mo-depleted conditions, the wild type strain as well as DJ33 (Δ*nifDK*) and UW356 (Δ*nifA*, encoding the *nif* gene transcriptional activator) exhibited concomitant production of ethylene and ethane diagnostic of alternative nitrogenase activity. Surprisingly, the Δ*vnfDGK* Δ*anfDGK* strains UW466 and UW463 exhibited similar ethylene and ethane production levels than vnf^+^anf^+^ strains (Table 2) despite the absence of Vnf proteins (Figure 5C). However, these strains accumulated high levels of NifDK. This observation suggests the plausible insertion of FeV-co into NifDK under Mo-depleted conditions, which would be responsible for the observed residual nitrogenase activities. No effect of the *mosAB* mutation on nitrogenase activity was detected in this condition in any of the tested genetic backgrounds.

## Discussion

The *A. vinelandii* MoSto protein is rather unique both in function and in amino acid sequence having no known homologs in protein databases. It has the capacity to accumulate massive amounts of Mo (over 100 atoms per MoSto), which would be a favorable trait to add to engineered nitrogen fixing plants expressing bacterial Mo-nitrogenase provided this Mo was directly available to nitrogenase FeMo-co biosynthesis. However, such a direct transfer had not yet been experimentally proven. Here we show that the Mo stored at MoSto is directly available for FeMo-co synthesis *in vitro* and that interaction of MoSto with FeMo-co biosynthetic proteins stimulates cofactor synthesis. The exact mechanism by which this interaction stimulates cofactor synthesis is unclear. Direct transfer of Mo from NifQ to the NifEN/NifH complex during FeMo-co biosyntesis *in vitro* has been reported (Hernandez et al., 2008). The similarities between the Mo-Fe-S clusters found in NifQ and in NifEN purified from a Δ*nifH* background (Soboh et al., 2006; George et al., 2007) support such direct connection. The expected primary path for molybdenum incorporation into FeMo-co would include molybdate transport, storage at MoSto, transfer to NifQ, and finally to the NifEN/NifH complex. However the results shown here indicate some degree of NifQ function replacement by MoSto in the *in vitro* system. Mo release from MoSto occurs spontaneously *in vitro* above pH 6.8 (Schemberg et al., 2008) and perhaps an interaction with NifEN facilitated Mo transfer. In this context, it is interesting to note that a NifH-independent pathway for Mo transfer into NifEN was previously postulated (Soboh et al., 2006). Thus, the ability of MoSto to donate Mo for FeMo-co synthesis might also be relevant *in vivo* and could underlie the phenotypic reversal of *nifQ^-^* by excess molybdate (Joerger and Bishop, 1988; Rodriguez-Quinones et al., 1993).

Mo release from MoSto and its utilization for *in vitro* for FeMo-co synthesis occurs at pH 7.5 in the presence of 1.23 mM ATP and an ATP regenerating enzyme in the reaction mixtures. It is known that MoSto catalyzed ATP-hydrolysis promotes formation of polyoxomolybdate clusters inside the MoSto cage while molybdate release from MoSto is favored at pH 7.5, but only after ATP is consumed (Poppe et al., 2018). Therefore, the buffer composition of the *in vitro* FeMo-co synthesis assay should preclude Mo release from MoSto. This discrepancy can be explained if FeMo-co biosynthesis and nitrogenase reconstitution alter the equilibrium of Mo binding/release by removing Mo from the available pool.

MoSto endows *A. vinelandii* with the ability to maintain high Mo-dependent diazotrophic growth rates under transient Mo limitation thus increasing strain competitiveness. It also confers certain degree of protection against W, a Mo antagonist that renders inactive molybdoenzymes. Normally W toxicity for nitrogen-fixing cells of *A. vinelandii* is evident when large excess of W over Mo (i.e., for W/Mo ratios > 150) is present in the culture medium (Keeler and Varner, 1957). This is not the case of the MoSto mutant, which exhibits high sensitivity to equimolar concentrations of M and W. Similar W sensitive phenotypes have been observed in mutants deficient in catechol siderophore production (Wichard et al., 2008). Both, Mo fluctuating conditions, including severe Mo limitation, and the presence of W at concentrations equal or higher than Mo, are environmentally relevant conditions. The average concentration of molybdate in terrestrial environments is 50 nM but its distribution is irregular (Hernandez et al., 2009). The advantage that MoSto might confer in the environment is however obscured under laboratory growth conditions in which large excess of molybdate is present in the medium.

Under Mo sufficient conditions more than 95% intracellular Mo is bound to MoSto. However, Mo-storage is not essential to achieve maximum nitrogenase activity as long as the growth medium contains excess molybdate (Figure 2B). This indicates that *A. vinelandii* ATP-dependent high affinity molybdate transport (Mouncey et al., 1995) is independent of the storage process and that molybdate uptake rates are enough to support maximum nitrogenase activity. Mo loading into MoSto is also an ATP-dependent process (Allen et al., 1999; Schemberg et al., 2008), which might impose an energy burden to the cell. This fact would explain why the mutant strain lacking MoSto is more competitive than the wild type under non-diazotrophic Mo-sufficient growth conditions. There are discrepancies in the literature as to whether or not MoSto expression is regulated by Mo. Pienkos reported constitutive MoSto expression (Pienkos and Brill, 1981) while Fenske found MoSto in cells grown at molybdate concentrations as low as 1 nM but not in Mo-free medium (Fenske et al., 2005). Our results are in line with constitutive expression since we were able to detect MoSto in cells grown in medium with Mo levels below an ICP-MS detection limit of 0.05 ppb. It is however possible that molybdate traces below our experimental detection limit are enough to induce MoSto expression.

We observed the co-existence of NifDK and VnfDGK polypeptides in cells grown under severe Mo-limiting or Mo-depleted conditions, although NifDK levels were much lower than those at standard Mo conditions (Figure 5). Transcripts of *nif* and *vnf* structural genes had been shown to co-exist at concentrations of Mo in the medium between 10 and 50 nM (Jacobson et al., 1986) but neither at 4 nM nor in Mo-depleted medium. It is likely that these discrepancies are due to the different sensitivity of Mo determination or product (either RNA or antigen) detection methods. Under Mo-deplete conditions the Δ*vnf* Δ*anf* mutants, lacking VnfDGK, appear to compensate with higher amounts of NifDK. It is know that the alternative nitrogenases can catalyze the reduction of acetylene by either two or four electrons to yield ethylene and ethane, respectively (Dilworth et al., 1988). Surprisingly, all strains exhibited activity with features typical of alternative nitrogenases under Mo-depleted conditions regardless of the presence of VnfDGK. Thus, simultaneous contributions from both systems cannot be ruled out. Hybrid Mo-nitrogenases carrying the cofactor of the V-nitrogenase (Moore et al., 1994) or even a biosynthetic precursor to FeMo-co (Soboh et al., 2010) have been generated *in vitro* and were shown to exhibit altered substrate specificities and product formation. Although in standard derepressing conditions the NafY protein seems to have a discriminating role in the insertion of Mo-nitrogenase active site metal cofactor (Rubio et al., 2004) the situation under extremely Mo-deficient conditions had not been tested.

In the absence of MoSto Vnf polypeptides are readily derepressed upon Mo step down, in contrast to the wild type that requires prolonged Mo starvation to initiate derepression (Figure 5B). Thus, the buffering effect of MoSto may also be important to maintain tight regulation of nitrogenase with different metal specificities.

## Conclusion

Under transient Mo-limiting conditions MoSto mutants showed low Mo accumulation levels, lost the ability to repress expression of the V-dependent nitrogenase, exhibited high sensitivity to W inhibition, and were less competitive than wild type in diazotrophic growth. Importantly, the *in vitro* FeMo-co synthesis assay establishes the donation of Mo from MoSto to FeMo-co biosynthetic proteins via direct interaction. MoSto provides robust Mo-dependent nitrogen fixation under Mo-limiting conditions to its prokaryotic host. A corollary to these results is the need to incorporate the MoSto genes into the prokaryotic gene complement required to engineer nitrogen-fixing plants (Allen et al., 2017; Buren et al., 2017; Buren and Rubio, 2018).

## Materials and Methods

### Generation of *A. vinelandii* Strains

Strains and plasmids used are listed in Table 3. *A. vinelandii* DJ (wild type) (Setubal et al., 2009), UW356 (*ΔnifA::spc*) (Poza-Carrion et al., 2014) and DJ33 (*ΔnifDK*) (Robinson et al., 1986) have been previously described. Strains UW394, UW466, and UW463 carrying in-frame deletions of the *mosAB*, *vnfDGK/anfDGK*, and *mosAB/vnfDGK/anfDGK* genes, respectively, were generated in this work. Deletions were incorporated into the *A. vinelandii* chromosome by transformation and gene replacement as described (Dos Santos, 2011).

**Table 3:** Bacterial strains and plasmids.

Strain or plasmid	Genotype	Resource
***Escherichia coli***		
DH5α	F– φ80ΔlacZM15 Δ(lacZYA-argF)U169 deoP recA1 endA1 hsdR17 (rK*^-^*mK^-^)	Sambrook and Russell, 2001
BL21(DE3)pLysS	F′- ompT gal[dcm][lon] hsdsB (rB^-^ mB*^-^*; an *E. coli* B strain) with DE3 and pLysS	Studier and Moffatt, 1986
***Azotobacter vinelandii***		
DJ	Highly transformable variant of OP	Setubal et al., 2009
UW394	*ΔmosBA::spc*	This study
UW466	*ΔvnfDGK::tet→ ; ΔanfDGK::kan→*	This study
UW463	*ΔmosBA::spc; ΔvnfDGK::tet→ ; ΔanfDGK::kan→*	This study
UW356	*ΔnifA::spc*	Poza-Carrion et al., 2014
DJ33	*ΔnifDK*	Robinson et al., 1986
**Plasmids**		
pBluescript KS (+)	Cloning vector	Agilent
pGEMT-vector	Cloning vector	Promega
pET28a (+)	Expression vector	Novagen
pUC4K	Vector containing Kan resistance cassette	Pharmacia
pBBR1-MCS3	Vector containing Tet resistance cassette	Obranic et al., 2013
pHP45Ω	Vector containing Spc/Sm resistance cassette	Prentki and Krisch, 1984
pRHB268	pGEMT carrying *mosBA* flanking regions and spc cassette	This study
pN2MN14	pBSKS(+) carrying *anfDGK* flanking regions and kan cassette	This study
pN2MN18	pBSKS(+) carrying *vnfDGK* flanking regions and tet cassette	This study
pN2MN72	*mosBA* genes cloned into pET28a (+)	This study


Strain UW394 was generated by transforming *A. vinelandii* DJ with plasmid pRHB268 in which the *mosBA* genes had been replaced by an spectinomycin resistance cassette obtained from pHP45Ω (Prentki and Krisch, 1984). pRHB268 is a derivative of pRHB266, which contains the *mosBA* region amplified by PCR using oligonucleotides 5′-CGCTCGCCCAGCTCGGTCAGGCGCA-3′ and 5′-CAGAGACCTGCTCGCCAGCTGAAATCC-3′ and cloned into pGEM-T. pRHB266 was digested with *BspE*I*/Age*I restriction enzymes to eliminate the *mosBA* genes, followed by Klenow treatment and blunt end ligation for the insertion of the *Sma*I-digested spectinomycin resistance cassette.

In-frame deletions of alternative nitrogenases were generated by co-introducing plasmids pN2MN14 and pN2MN18 into *A. vinelandii* DJ and UW394 to generate strains UW466 and UW463, respectively. To generate pN2MN14, DNA regions flanking *anfDGK* were amplified by PCR using oligonucleotides 5′-GGTTTCTCGAGATGACTCGTAAAGTAGCCAT-3′ and 5′-GATGGGATCCGACACATCTCCTTTAGAGTGA-3′ for the region upstream *anfD* and oligonucleotides 5′-ACCTGGATCCGGAAATGGACATCGAAGCCA-3′ and 5′-TACCTCTAGATGAGGACCCATTCCTTGTTC-3′ for the region downstream *anfK*. PCR products were digested with *Xho*I*/BamH*I*/Xba*I and cloned into *Xho*I and *Xba*I sites of pBlueScript KS (+) in a quadruple ligation reaction together with *BamH*I-digested kanamycin resistance cassette obtained from plasmid pUC4K by amplifying a PCR product using oligonucleotides 5′-AATTGGATCCGGGAAAGCCACGTTGTGTCTC-3′ and 5′-AATTGGATCCCTTTTGCTTTGCCACGGAACGG-3′. To generate pN2MN18, DNA regions flanking *vnfDGK* were amplified by PCR using oligonucleotides 5′-AGGCCTCGAGTGCATGACCGATGGGAC-3′ and 5′-CCATGGATCCGATTGAAGTCTCCTCGGCTCT-3′ for the region upstream *vnfD* and oligonucleotides 5′-GTGGTGGATCCAGGTGCCGGAGCGGTTTCC′-3′ and 5′-GGGTTCTAGAAGTCCAGGCGGACATGGC-3′ for the region downstream *vnfK*. PCR products were digested with *Xho*I*/BamH*I*/Xba*I and cloned into *Xho*I and *Xba*I sites of pBluescript KS (+) in a quadruple ligation reaction together with *BamH*I-digested tetracycline resistance cassette obtained from plasmid pBBR1-MCS3 by amplifying a PCR product using oligonucleotides 5′-CCGGGATCCCTCATGTTTGACAGCTTATCAT-3′ and 5′-CCGGGATCCGGAGTGGTGAATCCGTTAGC-3′ (Obranic et al., 2013).

Isolation of genomic DNA from *A. vinelandii* strains was performed by using DNAeasy^TM^ Tissue Kits (Qiagen). Generated *A. vinelandii* mutant strains were confirmed by PCR analysis and by immunoblot analysis with appropriate antibodies.

*Escherichia coli* DH5α was used for cloning procedures. Plasmid constructions, PCR DNA amplifications, and *E. coli* transformations were carried out by standard methods (Sambrook and Russell, 2001). Restriction analysis and DNA sequencing was used to confirm accuracy of all DNA constructs. To overexpress the *A. vinelandii* *mosBA* genes in *E. coli*, the *mosBA* genomic region of *A. vinelandii* was amplified by PCR using oligonucleotides 5′-GCGCGAATTCGCCAACTCGACAGCG-3′ and 5′-GCGCGCGGCCGCTCAGGCCGGACGCACA-3′, digested with *EcoR*I and *Not*I, and cloned into the *EcoR*I and *Not*I restriction sites of expression vector pET28a (+) to generate plasmid pN2MN72.

### Bacterial Strains and Growth Conditions

*Escherichia coli* DH5α was cultivated in Luria–Bertani medium at 37°C with shaking (250 r.p.m.). Antibiotics were added at standard concentrations (Sambrook and Russell, 2001). For MoSto overexpression experiments, *E. coli* BL21(DE3) pLysS strain was transformed with plasmid pN2MN72 and cultivated in 4 L fermentors in Luria–Bertani (LB) medium supplemented with 0.3 mM ammonium ferric citrate, 0.3 mM cysteine and, when indicated, 1 mM Na_2_MoO_4_. Fermentor cultures started at a cell OD_600_ of 0.022 and proceeded for 18 h at 30°C with air sparging (2.5 l/min) and stirring (300 r.p.m.).

*Azotobacter vinelandii* strains were cultivated in Burk’s modified medium (containing 28 mM ammonium acetate) or in Burk’s modified N-free medium at 30°C (Strandberg and Wilson, 1968) with modifications of metal contents (Mo, Fe, V, and W), when indicated. Antibiotics were added at standard concentrations (Curatti et al., 2005). Regarding Mo, three types of culture medium were used here and are defined as Mo-limited, Mo-depleted and Mo-standard. Mo-limited medium was prepared without molybdate and contained chemical components of high purity. All glassware used was acid washed and rinsed with milliQ water (Chatterjee et al., 1994). Mo-limited medium contained 2.1–4.4 nM Mo as determined by ICP-MS. Depletion of Mo traces still remaining in Mo-limited medium was achieved by incubating Mo-starved *A. vinelandii* DJ cells in 4 L of Burk’s modified N-free Mo-limited medium supplemented with 1 μM NaVO_3_. After 1 h of incubation, 2 L were collected and cells removed by centrifugation followed by filtration of the supernatant. The remaining 2 L of culture were supplemented with 28 mM ammonium acetate, incubated for 1 additional hour, after which cells were removed by centrifugation followed by filtration of the supernatant. Polypropylene plastic was used to harvest Mo-depleted medium. Collected medium remained sterile for months on the shelf. Mo levels in Mo-depleted medium were below the detection limit of the ICP-MS. Mo-depleted medium was used for competitive index analysis and the analysis of Anf*^-^* Vnf*^-^* strains. Mo-standard medium contained 1 μM Na_2_MoO_4_ and was prepared by standard procedures.

For nitrogenase derepression experiments, inoculum cultures were previously grown in Mo-limited Burk’s modified medium (Mo-starving conditions), in Mo-depleted Burk’s modified medium (Mo-depleted conditions), or in Burk’s modified medium (Standard conditions). Mo-starved inoculum cultures had been previously grown and transferred at least three times in Mo-limited Burk’s modified medium. *A. vinelandii* cells were then collected by centrifugation, washed with N-free Mo-limited medium and resuspended in N-free medium supplemented with 1 μM NaVO_3_ and lacking or containing Mo, as indicated in each experiment. Similar procedure using Mo-depleted medium was followed to obtain Mo-depleted inoculum cultures. Each derepression culture was inoculated at an optical density at 600 nm (OD_600_) of 0.3 and further cultivated for at least 4 h (8 h for *anf* *vnf* mutants) at 30°C with shaking (200 r.p.m.). At different times during derepression, culture samples were collected and subjected to the following analyses: determination of *in vivo* acetylene reduction activity, determination of cellular-bound molybdenum, growth as estimated by OD_600_, and detection of MoSto, NifDK, and VnfDGK proteins y immunoblot. Polypropylene filter tips were used for sample manipulation to prevent molybdenum traces.

For growth curves, *A. vinelandii* strains were cultured in NH_4_^+^-containing (nitrogenase repressing) solid Burk’s modified medium under Mo-limited or Standard conditions for at least 3 days. When Mo-starved cells were required, *A. vinelandii* strains were inoculated into Mo-limited Burk’s modified medium at least three times. Individual colonies were inoculated into liquid Burk’s modified medium and cultures were grown overnight to an OD_600_ ≈ 2. Inoculum cultures were then used to inoculate liquid N-free (nitrogenase derepressing) Burk’s modified medium with different Mo, V, and W contents: 1 μM NaVO_3_; 1 μM Na_2_MoO_4_ plus 1 μM NaVO_3_; 1 μM NaVO_3_ plus 1 μM Na_2_WO_4_; and 1 μM Na_2_MoO_4_ plus 1 μM NaVO_3_ plus 1 μM Na_2_WO_4_. Addition of NaVO_3_ permitted expression of V-nitrogenase under Mo-limited conditions. Non-diazotrophic growth conditions in Burk’s modified medium were analyzed as controls. Three hundred μl cultures were incubated at 30°C in 96-well plates at intensive speed setting for 48 h in a Bioscreen C apparatus (Thermo Fisher).

### Competitive Index Assay

Competitive index (CI) was defined as the mutant-to-wild-type ratio within the output sample, divided by the corresponding ratio in the inoculum (Macho et al., 2007). Precultures of wild type and UW394 were grown in Burk’s modified medium to exponential phase, mixed to a final OD_600_ of 0.1, and used to inoculate the Burk’s modified N-free medium as indicated in each experiment. Molybdenum and nitrogen regimes established in the precultures were maintained during co-growth in liquid medium. Twenty μl of serial dilutions of the mixed cultures were sampled at incubation times 0 and 22 h and plated onto solid Burk’s modified N-free medium and solid Burk’s modified N-free medium supplemented with spectinomycin (to inhibit growth of the wild type strain). Time 0 h determinations give input mutant to wild type ratios whereas time 22 h determinations give output CI values. Calculated CIs are the mean of three independent experiments with standard errors.

### *In vivo* Nitrogenase Activity Assays

*In vivo* nitrogenase activity was determined by the acetylene reduction assay (Stewart et al., 1967) with ethylene and ethane formation being analyzed in a Shimadzu GC-2014 gas chromatograph. Five ml culture samples were transferred to 26 ml vials sealed with rubber stoppers. After injection of 1.5 ml acetylene to each vial, reactions proceeded for 30 min at 30°C with shaking. Reactions were then stopped by addition of 0.1 ml of 8 M NaOH. To detect ethylene, 50 μl samples of the gas phase of each reaction assay were injected in a PoraPak N 80-100 column. Gas chromatography temperature conditions were: 150°C at the injection port; 90°C at the column and N_2_ carrier gas; and 150°C at the flame ionization detector (FID). To detect ethylene and ethane, 500 μl of the gas phase of each reaction assay were injected in a PoraPak N 50-80 column. Gas chromatography temperature conditions were: 180°C at the injection port; 60°C at the column and N_2_ carrier gas; and 200°C at the FID.

### Mo Determinations

Determination of Mo content in Mo-limited and Mo-depleted media was carried out by ICP-MS. Mo content of *A. vinelandii* cells was determined by Inductively Coupled Plasma Optical Emission Spectroscopy (ICP-OES) or by Inductively Coupled Plasma Mass Spectroscopy (ICP-MS). Cell culture samples (40 ml) were harvested by centrifugation at 5,000 r.p.m. for 5 min at 4°C, washed three times with Mo-limited or Mo-depleted medium, and collected under the same conditions. Pellets were dried at 100°C until ashes were formed and then resuspended in 5% nitric acid solution for ICP analysis. Culture samples were analyzed by ICP-OES at the ionomic service of the CEBAS-CSIC (Spain) or at ICP-MS at the Unit of Metal Analysis of the University of Barcelona Scientific and Technology Center (Spain) if Mo levels were too low to be detected by ICP-OES. Whole cell Mo contents are referred to as pmol Mo per cells contained in 1 ml of culture at an OD_600_ equal to 1 (2.2 × 10^8^ cells).

Mo determinations in purified MoSto preparations were carried out by ICP-OES or ICP-MS. The colorimetric method of Cárdenas (Cárdenas and Mortenson, 1974) was used to follow Mo-containing fractions during purification of MoSto from *A. vinelandii* cells.

### Protein Methods

Protein concentration was determined by the bicinchoninic acid method with BSA as the standard (Smith et al., 1985). Procedures for SDS-PAGE (Laemmli, 1970) and immunoblot analysis (Brandner et al., 1989) have been described. Protein samples for immunoblot analyses were prepared by mixing pelleted cells with 100 mM Tris-HCl at a final OD_600_ of 4, adding Laemmli buffer 4X supplemented with 0.1 M DTT, heating at 95°C for 3 min, and removing debris by centrifugation at 12000 r.p.m. for 2 min to obtain solubilized protein samples. Six μl samples were loaded per lane for NifDK and MosAB detection, 8 μl for VnfK detection and 15 μl for VnfG detection. After SDS-PAGE, proteins were transferred to nitrocellulose membranes and detected with specific antibodies against NifDK, MosAB, VnfK, or VnfG used at 1:2500 dilutions. MosAB polyclonal antibodies were produced in rabbit (CIB-CSIC). Secondary anti-rabbit Alkaline Phosphatase was used at 1:5000 dilution. NBT/BCIP (Nitroblue tetrazolium and 5-Bromo-4-chloro-3-indolyl phosphate) was used to develop immunodetection signal membranes. ImageJ software was used to quantify the protein levels in immunoblot membranes. The amount of MoSto protein in partially purified preparations from *A. vinelandii* cells was quantified against calibration curves generated with known amounts of pure MoSto obtained from recombinant *E. coli* cells.

### Purification of MoSto From *A. vinelandii* and Recombinant *E. coli* Cells

*Escherichia coli* BL21(DE3) pLysS pN2MN72 cell-free extracts were prepared by mixing cells in binding buffer 50 mM Na_3_PO_4_ pH 7.2 buffer, 500 mM NaCl, 10 mM imidazole in a ratio 1:2 and passing twice through a French Press (1,500 psi) followed by ultracentrifugation at 24,000 r.p.m. for 30 min at 4°C. Cell free extract is loaded into HiTrap Ni^+^ column (GE Healthcare) pre-equilibrated with binding buffer in a AKTA prime FPLC inside the glove box. The column was washed with binding buffer followed by two extra washes at 30 and 60 mM imidazole. Elution was carried out at 250 mM of imidazole. Eluted fractions containing purified MoSto were pooled, desalted and exchanged in 50 mM Na_3_PO_4_ pH 7.2 buffer, 500 mM NaCl, 10% glycerol. MoSto was stored in liquid nitrogen until used.

To purify MoSto from *A. vinelandii* cells, strain DJ was grown in 100-L fermentor with Burk’s modified medium containing 10 μMNa_2_MoO_4_ and 12.8 mM urea at 30°C for 20 h maintaining 3% dissolved O_2_. Cell-free extract preparations (French Press followed by cell debris removal by ultracentrifugation) and MoSto purification, including DEAE-Sephacel chromatography, ammonium sulfate fractionation, and Superdex-200 gel filtration were performed as described (Fenske et al., 2005). Fractions pooled and used for further chromatographic steps were selected according to their SDS-PAGE profiles and Mo determination results. MoSto was stored in liquid nitrogen until used.

### *In vitro* FeMo-co Synthesis and Nitrogenase Activation Assays

*In vitro* FeMo-co synthesis assays were carried out as described (Curatti et al., 2007). Reactions were carried out in acid-treated 9-ml serum vials sealed with serum stoppers under argon/acetylene (93%/7%) atmosphere. When indicated, MoSto purified either from *E. coli* or from *A. vinelandii* was added to the *in vitro* FeMo-co synthesis reactions as sole source of Mo. Activity of reconstituted NifDK was analyzed by the acetylene reduction assay after addition of excess NifH as described (Curatti et al., 2007). Ethylene formation was measured in a Shimadzu GC-2014 gas chromatograph equipped with a PoraPak N 80-100 column. The specific activity of each protein is defined as nanomoles of ethylene formed per minute per mg of NifDK protein.

Protein–protein interaction requirement for Mo donation by MoSto was addressed by inserting a 3-kDa pore-size cut-off dialysis membrane between a MoSto solution on one side and the mixture of FeMo-co biosynthetic proteins and apo-NifDK on the other side. FeMo-co synthesis and nitrogenase activation reactions were carried out at 30°C. Samples to quantify reconstituted nitrogenase activity were taken at 2, 10, and 30 min and analyzed for acetylene reduction.

## Author Contributions

MN-R and JB carried out the experimental work. MN-R and LR performed experimental design, data analysis, and wrote the manuscript.

## Conflict of Interest Statement

The authors declare that the research was conducted in the absence of any commercial or financial relationships that could be construed as a potential conflict of interest.
